# Multiple Polypoid Lesions in the Colon: What Is Your Diagnosis?

**DOI:** 10.5152/tjg.2020.19712

**Published:** 2022-08-01

**Authors:** Hakan Ümit Ünal, Alper Tunga Canpolat, Ali Türk, Murat Saruç

**Affiliations:** 1 Department of Gastroenterology, Acıbadem Mehmet Ali Aydınlar Universty Faculty of Medicine, İstanbul, Turkey; 2 Department of Internal Medicine, Bakırköy Acıbadem Hospital, İstanbul, Turkey; 3 Department of Radiology, Bakırköy Acıbadem Hospital, İstanbul, Turkey

## Question

### Case Presentation

**Figure 1.**
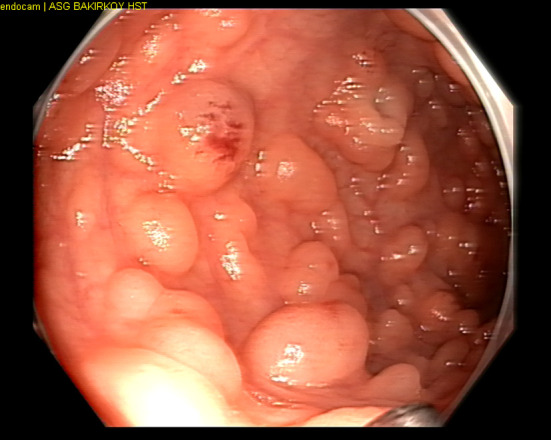
Polypoid lesions in the colon.

A 77-year-old female was admitted to our hospital because of aspiration pneumonia. She had been diagnosed with dementia before and had a permanent cardiac pacemaker. She had been bedridden for a year. Over the last 4 months, she had been fed via a nasogastric tube. She had been on metoprolol, levetiracetam, phenytoin, Aldactone, digoxin, and coumadin when she was admitted. According to her first biochemical test results, she had anemia due to iron deficiency (hemoglobin: 9.8 g/dL, hematocrit: 31.9%, white blood count: 11 000/µL, platelets: 359 000/µL, serum iron: 21 µg/dL (50-170), serum iron-binding capacity: 371 µg/dL). Based on these results, we planned a screening of the gastrointestinal system. In upper gastrointestinal endoscopy, there was erosive gastritis. On the other hand, there were a number of polypoid lesions whose diameter was changed between 5 and 15 mm from the proximal rectum to caecum. Those lesions were covered with normal colonic mucosa (Figure 1).

**In line with these “What would be your diagnosis for this case?”**

**Figure 2.**
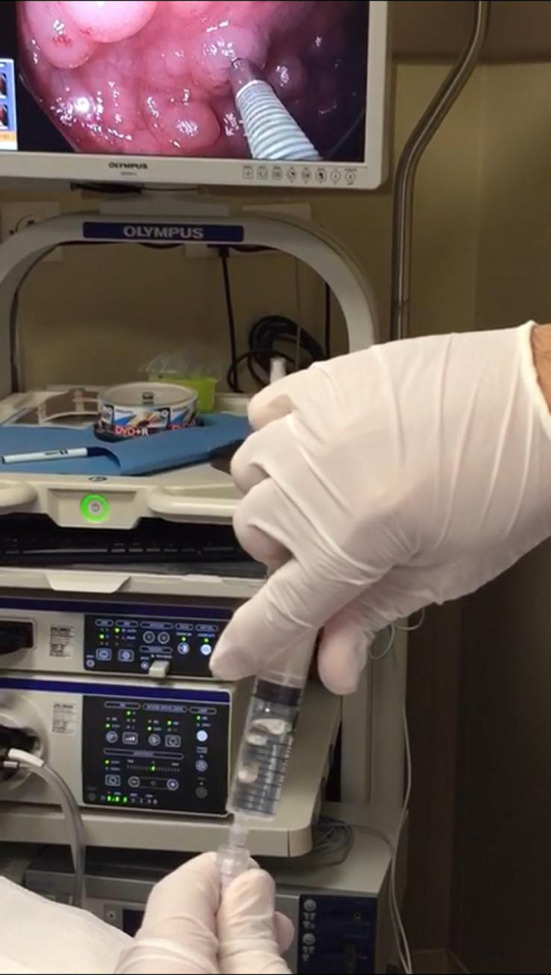
During the colonoscopy, aspiration of the polypoid lesions by using a sclerotherapy needle.

**Figure 3.**
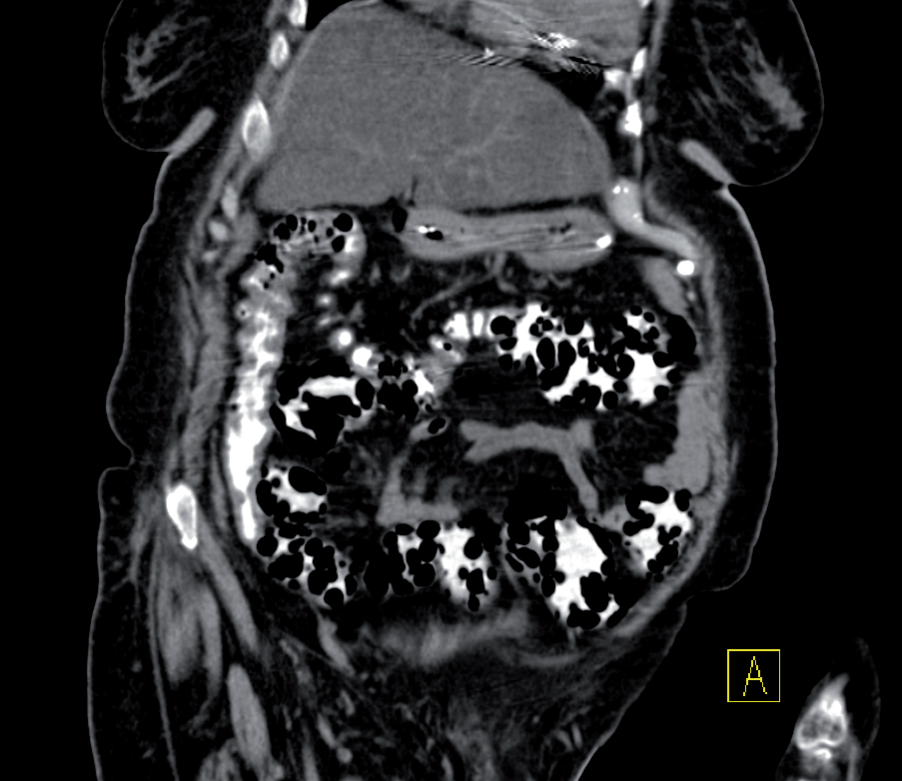
In computerized tomography, air-filled cysts in both submucosal and subserosal layers of the colonic wall.

During the colonoscopy, these lesions were thought to be pneumatosis intestinalis. We, therefore, aspirated some of these lesions by using a sclerotherapy needle. While we were aspirating the lesions, bubbles were formed in the injector filled with water up to half (Figure 2). Soon after the colonoscopy, computerized tomography was taken without contrast media, and we saw air-filled cysts in both submucosal and subserosal layers of the colonic wall. That’s why we diagnosed the patient with pneumatosis cystoides intestinalis (Figure 3).

## Discussion

Pneumatosis intestinalis (PI) refers to the presence of gas within the wall of the small or large intestine. Its clinical feature has varied from asymptomatic to life-threatening diseases such as gastrointestinal obstruction, perforation, and bleeding. Although its mechanism is not clear yet, some theories trying to explain etiopathogenesis have been put forward, such as mechanical pressure, infections, and biochemical theory, certain.

Pneumatosis intestinalis can accompany both primary gastrointestinal system diseases (such as intestinal ischemia, peptic ulcer, typhlitis, pseudomembranous colitis, and AIDS) and diseases other than gastrointestinal system diseases such as pulmonary diseases, scleroderma, diabetes. In addition, some endoscopic procedures and abdominal surgeries may trigger PI. Although plain abdominal graphy can help us to make a diagnosis, the most accurate diagnostic tool is computerized tomography. It enables us not only to make a diagnosis accurately but also to give us information about underlying diseases such as intestinal ischemia.

Normally, the diagnosis of PI is made using computed tomography. However, in our case, upon seeing the multiple polypoid lesions in colonoscopy, we aspirated them using a sclerotherapy needle and depicted the air. Thus, we confirmed the diagnosis before performing a computerized tomography.

In general, treatment is not necessary for this situation. Only if there are any underlying diseases or complications such as perforation should we manage it properly.

